# Total caffeine intake is associated with lower serum α-Klotho and may impact its detection accuracy: Evidence from NHANES 2007 to 2016 and molecular docking

**DOI:** 10.1097/MD.0000000000046592

**Published:** 2025-12-26

**Authors:** Fang-e Shi, Zhe Yu, Zhengyi Huang, Lingjie Cao, Cheng Chi, Jihong Zhu

**Affiliations:** a Department of Emergency, Peking University People’s Hospital, Beijing, China; b Department of Cancer Center, Beijing Ditan Hospital, Capital Medical University, Beijing, China; c Geriatric Department, Peking University People’s Hospital, Beijing, China.

**Keywords:** aging, biomarker, caffeine, NHANES, α-Klotho

## Abstract

The relationship between total caffeine intake (TCI) and aging remains understudied. α-Klotho (KL), a key aging-related circulating biomarker, is clinically valuable. Unlike prior studies focusing on coffee intake, we analyzed TCI (from diet and supplements) to isolate its independent association with KL and explore mechanisms via molecular docking. We thus conducted a cross-sectional analysis with NHANES 2007 to 2016 data to investigate TCI and serum KL levels in adults aged 40 to 79. Serum KL levels were determined using ELISA kits, while trained interviewers assessed TCI through 24-hour dietary recalls. Generalized linear regression models were employed to evaluate the correlation between TCI and serum KL levels. We utilized restricted cubic spline analysis to explore the dose–response relationship between the 2 factors. Subgroup analyses and molecular docking studies were also conducted to further understand the association. After adjusting for potential confounders, higher TCI was significantly associated with lower serum KL levels. In the fully adjusted model, compared to the lowest TCI group, KL levels decreased by −30.21 pg/mL (95% CI = −50.49 to −9.93 pg/mL) and −27.31 pg/mL (95% CI = −51.94 to −2.67 pg/mL) in the third and fourth groups, respectively, indicating a significant trend (*P* for trend = .005). Restricted cubic spline analysis revealed a linear dose–response relationship (*P* for nonlinearity = .362). Subgroup analyses showed that the negative correlation was more pronounced in participants aged < 60 years, those who were overweight/obese, and females. Molecular docking analysis suggested a direct interaction between caffeine and KL, with a binding energy of −5.299 kcal/mol, implying a stable interaction. These results suggest a potential pro-aging effect of caffeine. Further prospective studies and experimental validation are needed to clarify caffeine’s effects on KL. Notably, molecular docking indicates caffeine may interfere with α-Klotho immunoassay detection, emphasizing the need for caffeine abstinence before testing to enhance biomarker accuracy.

## 1. Introduction

With the enhancement of nutritional awareness and the increasing prominence of population aging issues, public attention to the connection between nutrition and aging has significantly increased. Caffeine, one of the most widely consumed dietary components in modern life, is present in a variety of foods and beverages, including coffee, tea, chocolate, and dietary supplements.^[1]^ Given that caffeine intake can produce significant physiological, metabolic, and psychological effects, it is crucial to thoroughly understand how regular caffeine consumption impacts human health, especially in terms of the aging process.^[2]^

Known research has highlighted numerous health benefits associated with coffee consumption. For example, caffeine intake has been found to have an inverse association with overall mortality and the risk of cardiovascular diseases (CVD).^[3,4]^ Coffee has also been shown to combat brain aging and delay the onset of Parkinson disease.^[5]^ However, the beneficial components in coffee responsible for its health benefits, particularly those related to delaying aging, remain unclear. Some studies indicate that decaffeinated coffee intake is linked with reduced overall mortality and cardiovascular mortality risk, suggesting that the health benefits of coffee may originate from other components.^[3,4]^ Research from Rutgers University points out that Eicosanoyl-5-hydroxytryptamine, a compound found in coffee, may help slow the progression of Parkinson disease and dementia.^[5]^ Researchers have also discovered that Trigonelline, found in coffee beans, is believed to aid in delaying the aging process.^[6]^ Moreover, the impact of coffee on telomere length varies across different types of coffee and studies, presenting conflicting results.^[7–9]^ Therefore, attributing health benefits such as delaying aging solely to caffeine is premature, and it is necessary to conduct separate studies on the effects of caffeine.

In the field of human aging research, the anti-aging protein klotho (KL) has garnered significant attention due to its involvement in the regulation of aging mechanisms – making it an ideal biomarker to dissect how caffeine (rather than coffee) influences aging processes. Early studies in mice with KL gene deficiency revealed premature aging phenotypes and shortened lifespans, whereas overexpression of the KL gene led to extended lifespans.^[10]^ KL comprises 3 main subtypes (α-KL, β-KL, and γ-KL),^[11]^ among which α-KL, also known as circulating KL, plays a role in blood, urine, and cerebrospinal fluid.^[12]^ KL is involved in regulating phosphate metabolism, mitigating oxidative stress, alleviating inflammatory responses, and modulating energy metabolism,^[12–14]^ and it also exhibits strong correlations with age-related metrics such as cognitive function and physical performance.^[12,15,16]^ Research has found that KL levels progressively decline after the age of 40, increasing susceptibility to a range of age-related conditions, including diabetes, AD, and chronic kidney disease.^[17–19]^ Moreover, dietary and lifestyle factors have been shown to influence KL protein expression. For instance, a pro-inflammatory diet, smoking, and alcohol consumption are associated with reduced KL levels, while adherence to the Mediterranean diet is linked to elevated KL levels,^[20–23]^ suggesting that nutrients may influence the aging process through their effects on KL levels.

Despite growing interest in caffeine’s role in aging and KL sensitivity to dietary factors, 3 critical gaps remain in the link between caffeine and KL: most studies^[24]^ use “coffee intake” as the exposure, which confounds caffeine with other coffee components and fails to capture non-coffee caffeine sources (e.g., tea, supplements); the dose–response relationship between total caffeine intake (TCI, not coffee) and KL remains unclear, with no linear/nonlinear patterns validated; no studies have explored whether caffeine directly interacts with KL, nor the implications for KL as a reliable aging biomarker. To address these gaps, we analyzed TCI from NHANES 2007 to 2016, complemented by molecular docking.

To address the aforementioned gaps, this study utilizes NHANES 2007 to 2016 data to: elucidate the association between TCI and circulating KL levels in adults aged 40 to 79, including the impact of covariates and dose–response patterns; explore potential caffeine-KL interactions via molecular docking to clarify mechanistic links and implications for KL as a reliable aging biomarker. This computational approach aims to predict the binding affinity and optimal binding modes of caffeine to KL, thereby providing preliminary insights into the putative mechanism underlying their relationship at the molecular level. Such structural analyses are expected to complement the epidemiological findings and offer a more comprehensive understanding of how caffeine may directly influence KL expression or function. It is anticipated that this approach will help address existing knowledge gaps in understanding the association between these 2 factors.

## 2. Materials and methods

### 2.1. Study population samples

NHANES is a robust cross-sectional survey by Centers for Disease Control and Prevention (CDC), assessing US health and nutrition status. Study details and participant recruitment are available on the official website of CDC. NHANES protocols are ethically approved, with participants’ written consent. For methodological details, the official NHANES website provides thorough information. NHANES selects samples via a multi-stage process involving primary sampling units, segments, households, and individuals, inviting around 12,000 individuals every 2 years across US counties, with variable response rates.

**Figure 1.**
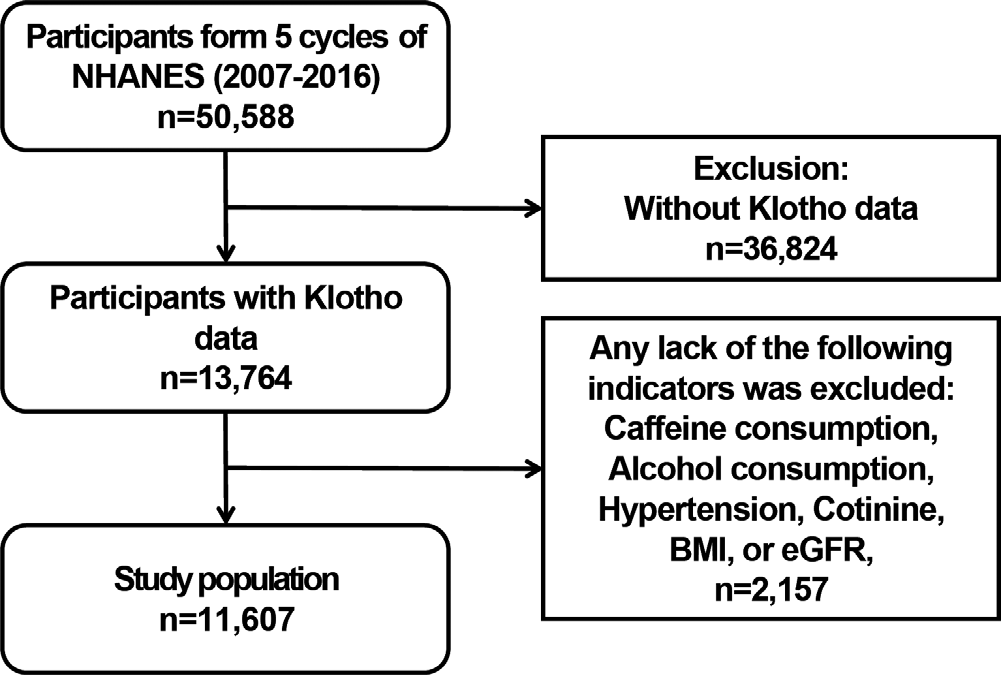
Flowchart of participant selection. BMI = body mass index, eGFR = estimated glomerular filtration rate.

For this study, 50,588 adults were enrolled from 5 cycles (2007–2016) of NHANES. Participants lacking serum KL level data (n = 36,824) were excluded from the analysis. Participants lacking caffeine intake data in two 24-hour dietary reviews and key covariate data (n = 2157) were also excluded from the analysis. Following the exclusion criteria and imputation procedures, the final cohort consisted of 11,607 participants, who were subsequently included in the analysis (Fig. 1). NHANES protocols were approved by the National Center for Health Statistics Ethics Review Board (protocol no. 2005-06).

### 2.2. Assessment of caffeine intake

TCI was assessed via two 24-hour dietary recalls to capture participants’ habitual dietary patterns – with the first conducted by trained staff at the Mobile Examination Center and a follow-up telephone interview 3 to 10 days later. Participants recorded all foods, beverages, and supplements consumed from midnight to midnight on the day before their in-person visit. This 2-recall design provided comprehensive data on daily consumption, ensuring TCI values reflect long-term habitual intake rather than short-term fluctuations.

To isolate caffeine as the independent exposure (distinct from other coffee components), we used the Food and Nutrient Database for Dietary Studies 5.0 to extract only caffeine content from all reported items. This step excluded coffee-specific bioactive compounds (e.g., chlorogenic acids, trigonelline) that could confound KL associations, thereby quantifying TCI rather than caffeine derived solely from coffee.

### 2.3. Determination of serum KL levels

Blood specimens were collected from NHANES participants aged 40 to 79 years who had provided consent for subsequent investigations. Participants were instructed to fast for at least 8 hours prior to blood collection to ensure consistent baseline conditions for the measurement of serum KL levels. The blood samples were processed promptly to minimize any potential degradation of analytes.

Serum KL levels were measured using commercially available ELISA kits manufactured by IBL International in Japan. The method employed, developed by Yamazaki et al, is a sandwich ELISA system specifically designed for measuring soluble α-KL levels in serum.^[25]^ This method has been rigorously validated from multiple angles.

All sample analyses were performed in duplicate according to the manufacturer’s protocol. The average of the tests was used to calculate the final value. Each result was reviewed to ensure compliance with laboratory standardized acceptability criteria before being reported. All pre-analyzed samples were stored at −80°C, and strict quality control measures were implemented throughout the experiment to maintain the integrity of the data.

### 2.4. Covariates

The selection of covariates was primarily guided by prior literature, considering the influencing factors and their associations with serum KL levels. Covariates primarily comprised: demographic variables: age, sex, income poverty ratio (PIR), ethnicity (non-Hispanic White, others), and educational level (below high school, high school or higher). Anthropometric measures: body mass index (BMI). Health behavior-related factors: alcohol consumption (<12 drinks annually, ≥12 drinks annually), dietary energy intake, and serum cotinine levels. Health status-related factors: hypertension and diabetes. Glomerular filtration rate, calculated using the Chronic Kidney Disease Epidemiology Collaboration (CKD-EPI) equation.^[26]^

### 2.5. Statistical analyses

TCI was log_2_ (n + 1) transformed to standardize the data and was analyzed both as a continuous and categorical variable. The relationship between TCI and KL levels was assessed using multivariate linear regression analysis.

As a continuous variable, the median TCI for each group was used, and a linear trend test was conducted. To facilitate interpretation, when examining standardized TCI as a continuous variable, we calculated the change in serum KL levels for each one standard deviation (SD) increase in the transformed TCI data.

For the analysis treating TCI as a categorical variable, we used one standard cup of coffee (8 ounces, containing 93 mg of caffeine^[27]^) as the reference and categorized TCI based on the following cutoffs: 50 mg (approximately 0.5 cups), 150 mg (approximately 1.5 cups), and 350 mg (approximately 4 cups), resulting in 4 groups. These cutoffs were chosen based on the following considerations: individuals consuming <0.5 cups per day are considered to have no regular coffee consumption; 1.5 cups is close to the average daily TCI for adults in the United States; 4 cups approach the upper limit of the recommended daily TCI (400 mg), reaching which adverse effects such as anxiety may occur. This categorization aligns with real-world caffeine consumption patterns.

Three regression models were utilized in this study: model 1, the baseline model without any covariate adjustments; model 2, the minimally adjusted model, which included age and sex as covariates; and model 3, the fully adjusted model, which expanded upon model 2 by incorporating all previously mentioned covariates. This fully adjusted model was used in subsequent subgroup analyses. We used the first group as the reference and determined the changes in serum KL levels for the remaining 3 groups.

To explore potential nonlinear trends, the dose–response relationship between TCI and serum KL levels for all participants was evaluated using restricted cubic spline (RCS) curves, with 4 knots placed at the 5th, 35th, 65th, and 95th percentiles.

According to previous literature, BMI, age, and sex are all significantly associated with serum KL levels. Subgroup analyses were conducted to delve deeper into the influence of these variables on serum KL levels. Additionally, linear regression models were constructed for each subgroup to examine the relationship between TCI and serum KL levels.

In the comparison of baseline characteristics between groups, categorical variables were presented as counts (percentages) and compared using the Chi-square test. Normally distributed continuous variables were presented as mean ± standard error and analyzed via analysis of variance. Skewed continuous variables were expressed as median (25th to 75th percentile) and compared using the Kruskal–Wallis test. To generate nationally representative estimates of the analysis results, we utilized the Mobile Examination Center weights recommended by the NHANES database. Data collection and statistical analyses were performed using R 4.3.3, with a significance threshold set at *P* < .05 (2-tailed).

### 2.6. Molecular docking

The binding affinities and interaction modes between caffeine and KL were analyzed using AutodockVina software (version 1.2.2; The Scripps Research Institute, La Jolla), a tool for computational protein-ligand docking.^[28]^ The structure of caffeine was obtained from PubChem Compound (National Center for Biotechnology Information, Bethesda ),^[29]^ while the 3D coordinates of KL (PDB ID: 5W21; resolution: 3.0 Å) were retrieved from the Protein Data Bank (PDB). Prior to docking analysis, all protein and ligand files underwent conversion to PDBQT format with exclusion of water molecules and addition of polar hydrogen atoms. The grid box was positioned to encompass the protein domain, allowing for molecular flexibility, with dimensions set at 30 Å × 30 Å × 30 Å and a grid point distance of 0.05 nm.

## 3. Results

### 3.1. Basic characteristics

Table 1 presents a summary of the clinical and biochemical data from the study cohort, which consisted of 11,607 participants, including 5517 (46.73%) males and 6090 (53.27%) females, with an average age of 56.42 ± 0.17 years, and the mean BMI was 29.58 ± 0.11 kg/m^2^. Among them, 84.79% of participants had received a college education or higher, and 75.16% were non-Hispanic White. The average daily TCI of the study participants was 153.00 (60.00–277.50) mg, with a median serum KL levels of 846.12 ± 5.26 pg/mL. Statistical differences were observed between groups of TCI and all characteristics except BMI. Participants demonstrating elevated TCI were more predisposed to manifest the following characteristics: male, Non-Hispanic White, higher educational attainment, higher income levels, hypertension, nondiabetic status, higher serum cotinine levels (indicative of smoking), higher energy intake, and more frequent annual alcohol drinking occasions, along with higher eGFR. Furthermore, participants with higher TCI also had lower serum KL levels.

**Table 1:** Based characteristics of all participants (n = 11,607).

Variables	Total	Grouping of TCI				Statistic	*P*
		G1	G2	G3	G4		
	11,607	3384	3506	3410	1307		
TCI, median (25th–75th), mg/d	153.00 (60.00, 277.50)	11.00 (1.50, 29.50)	97.50 (73.00, 123.50)	233.00 (187.50, 280.00)	470.00 (395.00, 597.50)	χ^2^ = 128,225.54	**<.001**
Klotho, mean (SE), pg/mL	846.12 (5.26)	874.39 (9.11)	853.50 (8.40)	830.70 (7.74)	827.83 (8.74)	*F* = 22.98	**<.001**
Age, mean (SE), yr	56.42 (0.17)	57.26 (0.26)	56.12 (0.26)	56.62 (0.25)	55.26 (0.29)	*F* = 15.40	**<.001**
Sex, n (%)							
Female	6090 (53.27)	1938 (59.70)	1981 (58.96)	1654 (50.73)	517 (40.35)	χ^2^ = 218.15	**<.001**
Male	5517 (46.73)	1446 (40.30)	1525 (41.04)	1756 (49.27)	790 (59.65)		
BMI, mean (SE), kg/m^2^	29.58 (0.11)	29.67 (0.20)	29.54 (0.18)	29.45 (0.15)	29.78 (0.22)	*F* = 0.00	.998
Race, n (%)							
Non-Hispanic White	5302 (75.16)	1013 (60.68)	1268 (66.67)	2015 (83.69)	1006 (90.94)	χ^2^ = 936.53	**<.001**
Non-Hispanic Black	2300 (8.90)	1062 (18.18)	783 (11.58)	396 (4.36)	59 (1.35)		
Mexican American	1741 (6.05)	613 (9.21)	607 (8.00)	423 (4.38)	98 (2.04)		
Other	2264 (9.89)	696 (11.93)	848 (13.76)	576 (7.57)	144 (5.68)		
Educational attainment, n (%)							
Below high school	3002 (15.21)	1059 (19.93)	997 (16.92)	682 (11.25)	264 (14.39)	χ^2^ = 102.62	**<.001**
High School or above	8605 (84.79)	2325 (80.07)	2509 (83.08)	2728 (88.75)	1043 (85.61)		
PIR, n (%)							
Not poor (PIR ≥ 1)	9648 (90.44)	2666 (86.21)	2903 (89.93)	2983 (93.36)	1096 (90.86)	χ^2^ = 95.45	**<.001**
Poor (PIR < 1)	1959 (9.56)	718 (13.79)	603 (10.07)	427 (6.64)	211 (9.14)		
Cotinine, Median (25th–75th), ng/mL	0.03 (0.01, 0.76)	0.02 (0.01, 0.10)	0.03 (0.01, 0.22)	0.03 (0.01, 1.63)	0.08 (0.01, 204.00)	χ^2^ = 65.63	**<.001**
Alcohol consumption, n (%)							
<12 drinks/yr	3193 (21.63)	1233 (32.97)	1064 (24.53)	704 (16.47)	192 (12.15)	χ^2^ = 375.67	**<.001**
≥12 drinks/yr	8414 (78.37)	2151 (67.03)	2442 (75.47)	2706 (83.53)	1115 (87.85)		
Diabetes, n (%)							
No	8854 (82.44)	2443 (78.65)	2654 (81.47)	2709 (84.44)	1048 (85.01)	χ^2^ = 47.73	**<.001**
Yes	2753 (17.56)	941 (21.35)	852 (18.53)	701 (15.56)	259 (14.99)		
Hypertension, n (%)							
No	4289 (42.81)	1102 (38.55)	1303 (42.19)	1342 (44.94)	542 (45.20)	χ^2^ = 31.70	**.001**
Yes	7318 (57.19)	2282 (61.45)	2203 (57.81)	2068 (55.06)	765 (54.80)		
eGFR, mean (SE), mL/min/1.73 m^2^	104.49 (0.28)	101.30 (0.47)	103.82 (0.42)	105.45 (0.34)	108.01 (0.38)	*F* = 144.67	**<.001**
Energy intake, mean (SE), kcal/d	2044.13 (11.38)	1864.85 (17.59)	1979.50 (19.63)	2109.48 (17.47)	2260.97 (28.13)	*F* = 166.36	**<.001**

The grouping of TCI ranges: G1: ≤ 50 mg/d; G2: >50 and ≤ 150 mg/d; G3: >150 and ≤ 350 mg/d; and G4: ≥350 mg/d. Bold indicates significance with *P* < .05.

BMI = body mass index, eGFR = estimated glomerular filtration rate, F = ANOVA, PIR = income poverty ratio, SE = standard error, TCI = total caffeine intake, χ^2^ = Chi-square test.

### 3.2. Association of caffeine consumption with serum KL levels

**Figure 2.**
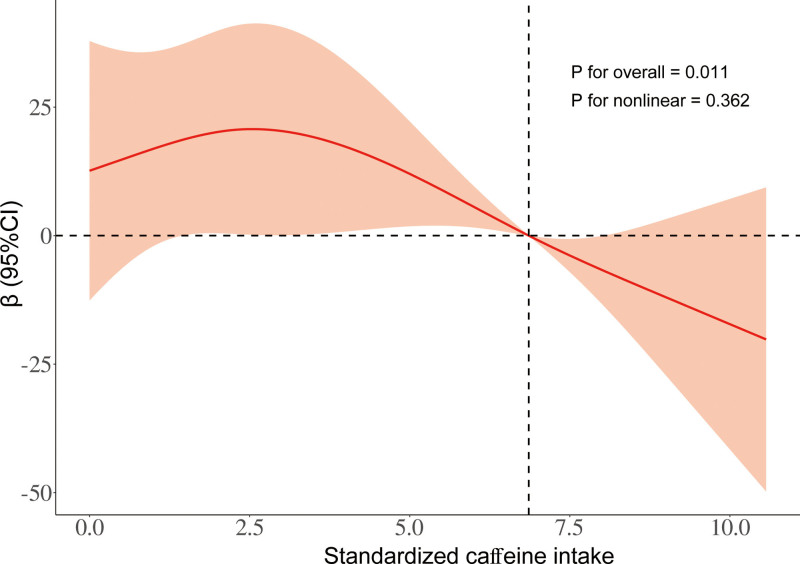
The linear relationship between standardized caffeine intake and the level of serum KL across the entire study population (*P*-value for nonlinearity = .362).

Table 2 shows the correlation between TCI and serum KL levels. We observed a significant correlation between the 2 variables, regardless of whether potential confounding factors were adjusted for. In model 1, which was unadjusted, every one SD increase in standardized TCI was associated with a change of −15.75 pg/mL (95% CI = −23.10 to −8.40 pg/mL, *P *< .001) in serum KL levels among all participants. After adjusting for age and sex in model 2, this association persisted, albeit slightly attenuated, with a change of −15.30 pg/mL (95% CI = −22.47 to −8.13 pg/mL, *P* < .001) per 1 SD increase in TCI. In model 3, however, upon further adjustment for all the covariates which mentioned early, the previously observed negative associations remained significant (change = −9.16 pg/mL, 95% CI = −16.95 to −1.38 pg/mL, *P* = .024). In the fully adjusted model, considering the first group of TCI as reference, participants in the third and fourth groups showed changes in serum KL levels of −30.21 pg/mL (95% CI = −50.49 to −9.93 pg/mL), and −27.31 pg/mL (95% CI = −51.94 to −2.67 pg/mL), respectively, indicating a significant trend (*P* for trend = .005). To further elucidate the dose–response relationship between TCI and serum KL levels, we employed a RCS function, depicted in Figure 2, and this analysis revealed a linear dose–response pattern between the 2 variables across all participants (*P* for nonlinearity = .362).

**Table 2:** The association between TCI and serum Klotho concentrations among all participants.

TCI	Klotho changes (pg/mL) and 95% CI					
	Model 1	*P*-value	Model 2	*P*-value	Model 3	*P*-value
Per SD increases	−15.75 (−23.10 to −8.40)	**<.001**	−15.30 (−22.47 to −8.13)	**<.001**	−9.16 (−16.95 to −1.38)	**0.024**
G1	Ref.	–	Ref.	–	Ref.	
G2	−20.89 (−43.57 to 1.79)	.075	−23.64 (−46.25 to −1.04)	**.044**	−20.38 (−42.79 to 2.04)	0.080
G3	−43.69 (−63.50 to −23.87)	**<.001**	−42.30 (−61.92 to −22.68)	**<.001**	−30.21 (−50.49 to −9.93)	**0.005**
G4	−46.56 (−69.82 to −23.29)	**<.001**	−45.19 (−68.00 to −22.37)	**<.001**	−27.31 (−51.94 to −2.67)	**0.034**
*P* for trend	−9.54 (−13.48 to −5.60)	**<.001**	−9.22 (−13.10 to −5.34)	**<.001**	−6.10 (−10.25 to −1.95)	**.005**

Model 1 was a crude model; model 2 was adjusted for age and sex; and model 3 was further adjusted for BMI, PIR, ethnicity, education attainment, serum cotinine, alcohol consumption, diabetes or not, hypertension or not, eGFR, and dietary energy intake. G1: ≤ 50 mg/d; G2: >50 and ≤ 150 mg/d; G3: >150 and ≤350 mg/d; and G4: ≥350 mg/d. Bold indicates significance with *P* < .05.

CI = confidence interval, SD = standard deviation, TCI = total caffeine intake.

### 3.3. Analysis stratified by subgroups

The study employed stratified analysis (Table 3). Adjustment for potential confounders was conducted, stratifying by BMI, age, and sex for serum KL levels and TCI. When TCI was considered as a categorical variable, compared to participants in the first group (G1), serum KL levels were significantly altered in other groups: they changed by −40.09 pg/mL (95% CI = −69.63 to −10.54 pg/mL) in the third group (G3) of middle-aged, and by −41.46 pg/mL (95% CI = −69.70 to −13.21 pg/mL) in the G3 of female. Additionally, in the fourth group (G4) of overweight/obese, serum KL levels changed by −35.53 pg/mL (95% CI = −61.47 to −9.59 pg/mL). A significant negative correlation between TCI and serum KL levels was observed in middle-aged, overweight/obese, and female subgroups, as evidenced by trend tests. However, with each SD increase in standardized TCI, the change in KL levels was −11.81 pg/mL (−22.90 to −0.73 pg/mL), *P* = .041, in the subgroup aged < 60 years; −7.14 pg/mL (−11.26 to −3.02 pg/mL), *P* = .001, in the overweight/obese subgroup; and −7.43 pg/mL (−13.31 to −1.55 pg/mL), *P* = .016, in the female subgroup. Significant linear negative correlation were observed in these 3 subgroups.

**Table 3:** The relationship between TCI and Klotho concentrations, stratified by age, BMI, and sex.

Participants	TCI	Klotho changes (pg/mL) and 95% CI	*P*-value
Age subgroup			
Age < 60 yr	Per SD increases	−11.81 (−22.90 to −0.73)	**.041**
	G1	Ref.	–
	G2	−33.83 (−65.74 to −1.92)	**.042**
	G3	−40.09 (−69.63 to −10.54)	**.010**
	G4	−29.90 (−66.11 to 6.30)	.110
	*P* for trend	−7.32 (−13.46 to −1.18)	**.022**
Age ≥ 60 yr	Per SD increases	−5.98 (−15.75 to 3.79)	.33
	G1	Ref.	–
	G2	−0.51 (−27.88 to 26.86)	.971
	G3	−18.14 (−41.76 to 5.47)	.137
	G4	−29.67 (−62.24 to 2.89)	.079
	*P* for trend	−4.70 (−9.43 to 0.02)	.055
BMI subgroup			
BMI < 25 kg/m^2^	Per SD increases	−6.72 (−25.28 to 11.84)	.480
	G1	Ref.	–
	G2	−12.14 (−53.55 to 29.28)	.568
	G3	−24.06 (−71.70 to 23.59)	.326
	G4	3.95 (−61.81 to 69.72)	.907
	*P* for trend	−2.69 (−12.87 to 7.48)	.606
BMI ≥ 25 kg/m^2^	Per SD increases	−9.63 (−16.89 to −2.36)	**.012**
	G1	Ref.	–
	G2	−22.27 (−47.43 to 2.88)	.087
	G3	−32.51 (−51.87 to −13.16)	**.002**
	G4	−35.53 (−61.47 to −9.59)	**.009**
	*P* for trend	−7.14 (−11.26 to −3.02)	**.001**
Sex subgroup			
Male	Per SD increases	−8.24 (−19.28 to 2.81)	.149
	G1	Ref.	–
	G2	−5.82 (−38.09 to 26.46)	.725
	G3	−16.22 (−48.80 to 16.37)	.333
	G4	−27.75 (−64.22 to 8.72)	.141
	*P* for trend	−4.76 (−11.49 to 1.97)	.170
Female	Per SD increases	−10.13 (−22.47 to 2.22)	.113
	G1	Ref.	–
	G2	−29.96 (−55.13 to −4.78)	**.023**
	G3	−41.46 (−69.70 to −13.21)	**.005**
	G4	−22.44 (−63.71 to 18.82)	.290
	*P* for trend	−7.43 (−13.31 to −1.55)	**.016**

The grouping of TCI ranges: G1: ≤ 50 mg/d; G2: >50 and ≤ 150 mg/d; G3: >150 and ≤ 350 mg/d; and G4: ≥350 mg/d. Bold indicates significance with *P* < .05.

BMI = body mass index, CI = confidence interval, SD = standard deviation, TCI = total caffeine intake.

### 3.4. Affinity assessment between caffeine and KL

To evaluate the binding affinity of caffeine with KL, molecular docking analysis was performed using AutodockVina (v.1.2.2). Docking poses and interactions between caffeine and KL were generated, and the binding energy for their interaction was computed. Results demonstrated that caffeine established noticeable hydrogen bonds with KL. Remarkably, caffeine displayed a binding energy of −5.299 kcal/mol with KL, indicating a remarkably stable binding interaction.

**Figure 3.**
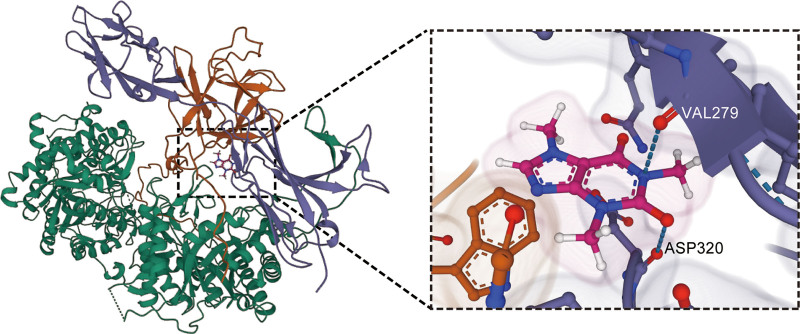
The docking results of KL with caffeine.

Caffeine forms hydrogen bonds with the VAL279 and ASP320 residues of KL (Fig. 3), which are likely part of the epitopes recognized by the capture antibody in ELISA kits. This suggests that caffeine may potentially block antibody binding, leading to an underestimation of the actual serum concentration of KL – a potential methodological artifact that has not been previously accounted for in existing studies. Moreover, this stable binding is consistent with the linear dose–response relationship identified by RCS analysis, suggesting that the effect of caffeine on KL may involve both “direct binding interference” and “dose dependency” – addressing both Research Gap (2) and (3) outlined in the Introduction.

## 4. Discussion

This study investigated the relationship between TCI and serum KL levels in a large, nationally representative sample of American adults aged 40 to 79. Our findings revealed that higher TCI was associated with lower serum KL levels among American adults, suggesting a potential pro-aging effect of caffeine. Even after adjusting for various potential confounding factors, although the statistical impact of TCI on KL levels was relatively weak, a significant negative correlation still existed. This result enhances our understanding of the relationship between caffeine consumption and aging processes.

Some studies have reported similar findings. For example, a study utilizing the NHANES database found an inverse relationship between caffeine intake and telomere length, suggesting that caffeine may contribute to cellular aging.^[7]^ Qush et al found that caffeine-containing diets may have negative impacts on gut environment and immunity.^[30]^ Additionally, Lopez-Garcia et al observed an inverse relationship between caffeine intake and all-cause mortality, which was also seen with decaffeinated coffee, indicating that the beneficial components in coffee contributing to these effects may be factors other than caffeine, possibly even overriding any adverse effects of caffeine.^[31]^ However, some studies have reported conflicting results. For instance, Tao et al found that caffeine can enhance telomerase reverse transcriptase (TERT) expression, leading to elongated telomeres and potentially delaying cellular aging.^[8]^ These conflicting findings highlight the complexity of the relationship between caffeine and aging, and suggest the need for further exploration of the interaction mechanisms between caffeine and KL.

Our study differs from Tang research^[24]^ in 3 critical ways: exposure variable: we analyzed TCI (diet + supplements, n = 11,607) covering non-coffee consumers, whereas Tang et al focused on coffee intake (n = 9811), confounding caffeine with other components; dose–response pattern: we observed a linear association (RCS *P* = .362) consistent with direct caffeine-KL binding, while Tang et al reported an L-shaped curve (likely due to coffee’s other components); mechanistic and practical implications: our molecular docking identified potential interference with KL immunoassays, leading to a novel recommendation (caffeine abstinence before testing), which Tang et al did not address. These differences highlight that our findings more accurately reflect caffeine’s independent effects on KL.

KL, a transmembrane protein, plays a pivotal role in regulating intracellular and extracellular calcium ion balance, antioxidative stress, anti-inflammation, and promoting cellular apoptosis. Abundant evidence suggests a close correlation between KL expression levels and aging as well as longevity.^[32]^ The negative correlation between TCI and serum KL levels may be due to caffeine’s ability to influence KL expression or stability. Currently, there is no direct evidence demonstrating the mechanism by which caffeine affects KL. This is one of the reasons we conducted molecular docking studies. However, we can analyze potential molecular mechanisms based on known regulatory pathways of KL. Studies have shown that KL expression levels may be regulated by signaling pathways such as Wnt/β-catenin, IGF1, and mTOR, and can also be influenced by oxidative stress, inflammatory responses, DNA methylation, and histone modifications.^[32]^ Caffeine primarily exerts its effects by influencing adenosine receptors, calcium ion channels, and mitochondrial function, and there may be complex crosstalk between these mechanisms and the regulation of KL. Notably, research has found that blocking adenosine receptors can inhibit oxidative stress and inflammatory responses,^[33]^ and caffeine has been linked to the induction of abnormal methylation.^[34]^ These mechanisms may be important ways by which caffeine regulates KL expression.

Moreover, molecular docking studies predict a potential direct interaction between caffeine and KL, this direct interaction suggests 2 non-mutually exclusive possibilities: caffeine may modulate KL biological function (e.g., by altering its stability or signaling activity), or caffeine may interfere with KL detection in serum assays. The direct binding observed here raises the hypothesis that caffeine could mask antibody recognition sites or induce conformational changes in KL, leading to underestimation of its actual concentration. This would imply that the observed negative association might reflect a methodological artifact related to biomarker detection rather than a true biological reduction in KL-mediated longevity pathways.

Notably, KL is widely regarded as a surrogate marker of health status and lifespan, but its utility as a biomarker depends on the reliability of its measurement. Our findings highlight a potential confounder: caffeine intake at the time of blood sampling may introduce bias in KL quantification. If validated, this would suggest that standardizing caffeine abstinence (e.g., 24 hours prior to testing) could improve the accuracy of KL as a clinical or research biomarker, particularly in populations with high caffeine consumption.

Further studies are needed to disentangle these possibilities, including in vitro experiments to assess whether caffeine affects KL immunoassay performance and longitudinal analyses to clarify whether caffeine-associated reductions in measured KL correspond to adverse health outcomes. For now, our data urge caution in interpreting KL-caffeine associations as evidence of caffeine’s direct impact on lifespan, emphasizing instead the need to account for caffeine exposure when using KL as a biomarker.

From a clinical perspective, our findings suggest that serum KL measurement – currently used in aging and chronic kidney disease research^[35,36]^ – should standardize caffeine abstinence (e.g., 24 hours prior to blood sampling), especially in populations with high caffeine intake (e.g., <60 years, overweight/obese). This would improve the reliability of KL as a biomarker, a practical advance lacking in previous literature.

We found that numerous confounding factors can influence KL levels, beyond the over a dozen factors we controlled for in our analysis. Other factors, such as diet, lifestyle, and genetic predispositions, may also play a role in modulating serum KL levels. For example, physical exercises and certain therapeutic drugs (e.g., ACE inhibitors, metformin, statins) have been shown to elevate circulating KL levels.^[37–40]^ Therefore, it is necessary to conduct reasonable subgroup analyses for covariates with high impact. The main strong correlates are age, BMI, and sex, which are the primary variables in our subgroup analysis.^[41,42]^ Our subgroup analysis revealed that the negative correlation between TCI and serum KL levels was more pronounced in participants aged < 60 years, those who were overweight/obese, and females. These findings suggest that the impact of caffeine on serum KL levels may vary across different demographic and physiological characteristics. The sensitivity observed in the female subgroup may be associated with the regulation of caffeine metabolism by estrogen^[43]^; the observed significant association in the overweight/obese population may be attributed to enhanced inflammatory status,^[44]^ which in turn affects caffeine metabolism. These subgroups may be more susceptible to the aging effects of caffeine, highlighting the need for tailored interventions and further research to understand the underlying mechanisms.

This study’s strengths include its large, nationally representative sample size. Additionally, the analysis accounted for a wide range of potential confounding factors, including socioeconomic status and personal behaviors. The data gathering process was conducted by NHANES researchers who had received proper training, ensuring the reliability of the measurements.

However, the study is subject to several limitations. Its cross-sectional design precludes the establishment of causal relationships. The population of middle-aged and elderly individuals consuming caffeine may represent a group with distinct lifestyles. TCI was predominantly self-reported, which could introduce recall bias. Moreover, the study did not control for all chronic diseases that may affect serum KL levels, such as respiratory, circulatory, urinary, and nervous system disorders. The impact of long-term medication on serum KL levels remains unclear, and the observed relationships may be influenced by these factors. The use of molecular docking also has limitations. While the method provide valuable insights into potential interactions, they do not account for the complex cellular environment and dynamic interactions that occur in vivo. Experimental validation is necessary to confirm the predicted interactions. Techniques such as surface plasmon resonance, isothermal titration calorimetry, and pull-down assays can be used to verify the binding affinity and specificity of caffeine to Klotho.

Understanding the mechanisms underlying the association between TCI and serum KL levels is crucial for developing targeted interventions. Mediation analysis can help identify the specific pathways through which caffeine affects KL expression and stability. Future studies should focus on longitudinal designs to establish causality, investigate the molecular mechanisms by which caffeine affects KL expression, and explore the potential benefits of reducing TCI in specific subgroups.

## 5. Conclusion

This study investigated the association between TCI and serum KL levels in 11,607 U.S. adults aged 40 to 79 using NHANES 2007 to 2016 data, complemented by molecular docking to explore underlying mechanisms. After adjusting for covariates, higher TCI was significantly associated with lower serum KL levels, with a linear dose–response relationship (*P* for nonlinearity = .362); this negative association was more pronounced in participants aged < 60 years, overweight/obese individuals, and females. Molecular docking further revealed that caffeine binds stably to KL at residues VAL279 and ASP320, suggesting caffeine may interfere with KL detection accuracy.

These findings imply a potential link between TCI and reduced KL, while also highlighting a critical methodological consideration: standardizing caffeine abstinence before KL testing may improve the reliability of KL as an aging biomarker. Future prospective studies should validate the causal relationship between TCI and KL, and experimental studies (e.g., surface plasmon resonance) are needed to confirm caffeine’s interference with KL immunoassays. Additionally, subgroup-specific interventions (e.g., targeted caffeine intake guidance for middle-aged females or overweight adults) warrant exploration.

## Acknowledgments

We extend our heartfelt appreciation to all the participants of NHANES for their invaluable contributions, as well as to the NHANES team for their unwavering dedication and commitment.

## Author contributions

**Conceptualization:** Fang-e Shi.

**Data curation:** Zhe Yu, Zhengyi Huang.

**Funding acquisition:** Jihong Zhu.

**Methodology:** Fang-e Shi, Zhe Yu.

**Project administration:** Cheng Chi.

**Resources:** Zhe Yu, Zhengyi Huang.

**Supervision:** Cheng Chi.

**Visualization:** Zhe Yu, Zhengyi Huang.

**Writing – original draft:** Fang-e Shi, Zhe Yu, Zhengyi Huang, Lingjie Cao.

**Writing – review & editing:** Fang-e Shi, Zhe Yu, Cheng Chi, Jihong Zhu.
